# Cytomegalovirus colitis presenting with lower gastrointestinal bleeding following chimeric antigen receptor‐T cell therapy

**DOI:** 10.1111/jcmm.18538

**Published:** 2024-07-19

**Authors:** Jabour Halloun, Itay Maza, Anat Stern, Israel Henig, Luiza Akria, Yaniv Zohar, Tsila Zuckerman, Ofrat Beyar‐Katz

**Affiliations:** ^1^ Department of Hematology and Bone Marrow Transplantation Rambam Health Care Campus Haifa Israel; ^2^ Gastroenterology Unit Rambam Health Care Campus Haifa Israel; ^3^ The Ruth and Bruce Rappaport Faculty of Medicine, Technion Haifa Israel; ^4^ Division of Infectious Diseases Rambam Health Care Campus Haifa Israel; ^5^ Hematology and blood bank, Galil Medical Center Nahariya Israel; ^6^ Faculty of Medicine in the Galilee Bar Ilan University Safed Israel; ^7^ Department of pathology Rambam Health Care Campus Haifa Israel

A 47‐year‐old female, was diagnosed with double hit large B‐cell lymphoma (LBCL). A PET‐CT scan at the time of diagnosis revealed extensive disease involvement in multiple areas, including the intestines, rectum, portal system, spleen, kidneys, urinary tract, as well as the presence of ascites. She was treated initially with dose‐adjusted EPOCH‐R (etoposide, prednisone, vincristine, cyclophosphamide, doxorubicin, and rituximab) but following 3 courses she was considered refractory. At that point she was referred to chimeric antigen receptor‐T cell (CAR‐T) therapy and underwent mononuclear apheresis for Axicabtagene ciloleucel production. Cell blood counts were normal, and no bone marrow involvement was detected based on the PET‐CT scan at diagnosis and prior to CAR‐T cell infusion. Before lymphodepletion, she was diagnosed with central nervous system (CNS) involvement. As a result, she received dexamethasone 10 mg twice daily before conditioning therapy. The dosage of steroids was gradually tapered during lymphodepletion therapy and her CAR‐T hospitalization. Cytokine release syndrome (CRS) grade I was observed on day +4, and she received one dose of tocilizumab on day +7 due to persistent fever. No immune effector cell‐associated neurotoxicity syndrome (ICANS) was observed.

On day +12 from CAR‐T cell administration, the patient developed hematochezia with hemodynamic instability and decreased haemoglobin levels of 2 g/dL from baseline blood counts. Notably, at that time point platelet counts were 136,000 cells/μL and absolute neutrophil count were 4600 cells/μL, showing no signs of early immune effector cell‐associated hematotoxicity (ICAHT). Lymphocyte values and inflammation markers are presented in Figure S1. A CT angiography showed active arterial bleeding in the right colon (Figure 1A). Since the patient had severe diarrhoea for 3 days, it was decided to move forward with colonoscopy immediately following the CT scan, without bowel cleansing. On colonoscopy, bowel prep was overall good, revealing numerous linear and deep ulcers throughout the entire length of the colon. In the right colon, a visible vessel with active oozing of blood was noted. The vessel was treated with a single Hemo‐clip, with satisfactory hemostatic result (Figure 1B). Pathology report showed colonic mucosa with ulceration and inflamed granulation tissue with no evidence of lymphoma. Notably, a few cells were positive for CMV (cytomegalovirus) immunostaining (Figure 1C). At the same time, peripheral blood showed CMV viral load of 20,000 copies/mL. Therefore, ganciclovir was introduced on day +20 of hospitalization with good clinical and biochemical response by day +29. After completion of anti‐CMV treatment, repeated colonoscopy showed mucosal scarring, without any significant ulcers or bleeding (Figure 2D). Timeline is presented in Figure 2.

**FIGURE 1:**
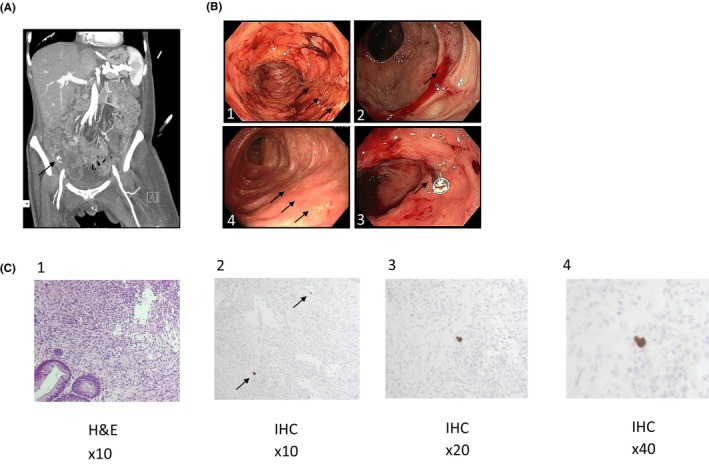
(A) CT angiography—showing active bleeding in the right colon. (B) Colonoscopy findings. (1) linear ulcer with few blood clots in the right colon, similar ulcers were noted throughout the entire colon. (2) visible vessel with active bleeding in the right colon, compatible with findings on CT angiography, (3) bleeding controlled by hemostatic clip, (4) linear ulcer healing following CMV treatment. (C) Histological findings from colon. (1) Histology obtained from colon (magnification × 10). (2) IHC staining for CMV (magnification × 10). (3) IHC staining for CMV (magnification × 20). (4) IHC staining for CMV (magnification ×40).

**FIGURE 2:**
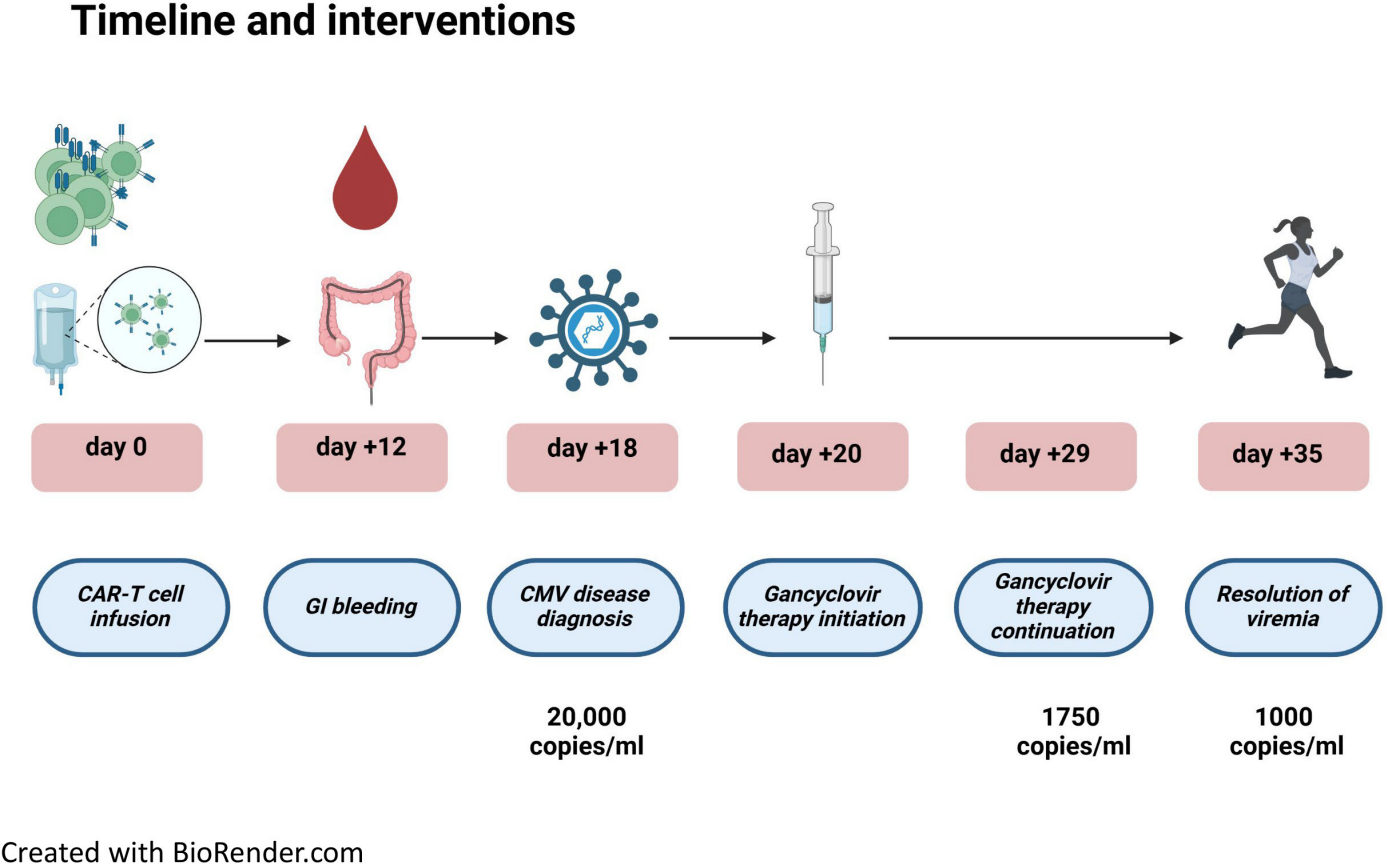
Timeline and interventions.

CMV reactivation is frequently observed in patients following CAR‐T cell therapy, with some cases resolving without anti‐viral therapy; however, CMV disease is rarely reported.^1^, ^2^ Certain risk factors associated with an increased risk of CMV reactivation in this population include steroid treatment or evidence of CMV replication prior to infusion.^3^, ^4^ Currently, there is no consensus on whether to screen regularly for CMV reactivation or to check CMV status only for high‐risk populations, resulting in each center using its own protocols. Furthermore, the therapeutic approach regarding CMV prophylaxis or pre‐emptive therapy in these patients remains unknown. In our center we routinely screen all CAR‐T patients for CMV reactivation 2 weeks following CAR‐T cell infusion.

There is one case described in the literature of CMV enteritis following CAR‐T cell therapy. The patient developed jejunal involvement, which resolved following foscarnet treatment but was later complicated by GI perforation and bleeding.^2^

Notably, autoinflammatory colitis mimicking inflammatory bowel disease (IBD) has been reported following CAR‐T cell therapy, and infections such as CMV colitis must be excluded to confirm this diagnosis.^5^

The current case underscores the importance of CMV analysis in patients treated with CAR‐T cells, particularly those with identified risk factors. The presentation of hematochezia, uncommon in CMV colitis, was the initial symptom in our case. Our experience highlights the criticality of early diagnosis and prompt initiation of antiviral therapy, leading to a favourable outcome.

## AUTHOR CONTRIBUTIONS

**Jabour Halloun:** Conceptualization (equal); writing – original draft (equal). **Itay Maza:** Conceptualization (equal); writing – original draft (equal); writing – review and editing (equal). **Anat Stern:** Conceptualization (equal). **Israel Henig:** Conceptualization (equal). **Luiza Akria:** Conceptualization (equal). **Yaniv Zohar:** Conceptualization (equal). **Tsila Zuckerman:** Conceptualization (equal). **Ofrat Beyar‐Katz:** Conceptualization (equal); writing – original draft (equal); writing – review and editing (equal).

## FUNDING INFORMATION

No funding for this study.

## CONFLICT OF INTEREST STATEMENT

The authors declare no conflicts of interest.

## Supporting information


Figure S1.

## Data Availability

The data that support the findings of this study are available upon request from the corresponding author.
