# Supplementary figures and images for: Cytomegalovirus colitis presenting with lower gastrointestinal bleeding following chimeric antigen receptor‐T cell therapy
Source: J Cell Mol Med. 2024 Jul 19;28(14):e18538. doi: 10.1111/jcmm.18538 (PMC11258881; doi:10.1111/jcmm.18538)

## Slide 1
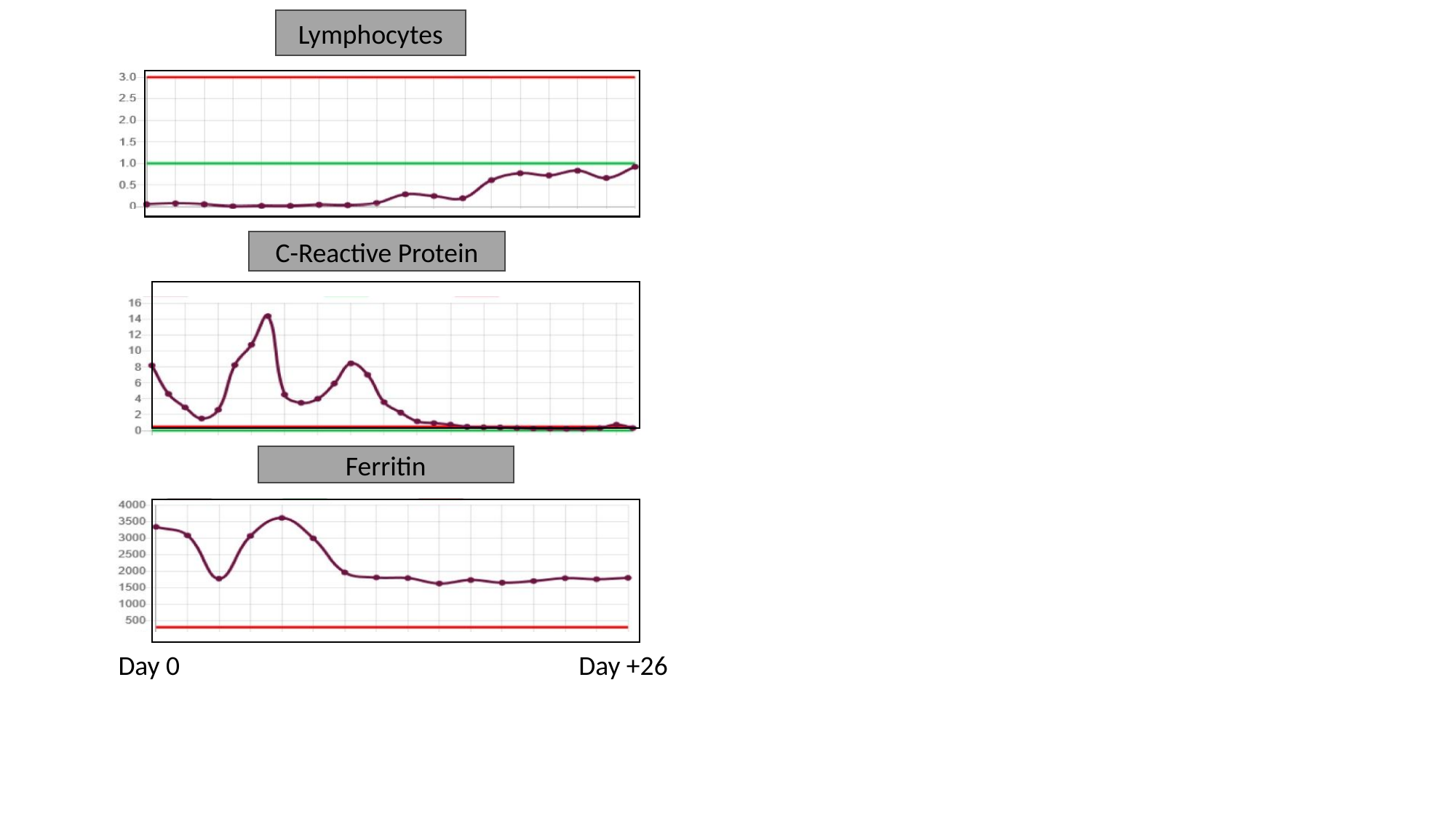

Lymphocytes
C-Reactive Protein
Ferritin
Day +26
Day 0

Supplement: Supplementary file 1 — Figure S1. [file JCMM-28-e18538-s001.pptx]
